# MAP3K4 kinase action and dual role in cancer

**DOI:** 10.1007/s12672-024-00961-x

**Published:** 2024-04-03

**Authors:** Yuxin Huang, Guanwen Wang, Ningning Zhang, Xiaohua Zeng

**Affiliations:** 1 Department of Breast Cancer Center, Chongqing University Cancer Hospital, School of Medicine, Chongqing University, Chongqing, China; 2 Department of Breast Cancer Center, Chongqing University Cancer Hospital, Chongqing, China

**Keywords:** MAP3K4, MAPK pathways, Binding partners, Organism development, Cancer

## Abstract

It is commonly known that the MAPK pathway is involved in translating environmental inputs, regulating downstream reactions, and maintaining the intrinsic dynamic balance. Numerous essential elements and regulatory processes are included in this pathway, which are essential to its functionality. Among these, MAP3K4, a member of the serine/threonine kinases family, plays vital roles throughout the organism's life cycle, including the regulation of apoptosis and autophagy. Moreover, MAP3K4 can interact with key partners like GADD45, which affects organism's growth and development. Notably, MAP3K4 functions as both a tumor promotor and suppressor, being activated by a variety of factors and triggering diverse downstream pathways that differently influence cancer progression. The aim of this study is to provide a brief overview of physiological functions of MAP3K4 and shed light on its contradictory roles in tumorigenesis.

## Introduction

Mitogen-activated protein (MAP) kinase signaling pathways are pervasive and highly stable biological processes in eukaryotic cells. These pathways facilitate collaborative and convergent responses to a broad spectrum of stimuli via protein kinase-type receptors, leading to significant cellular physiological manifestations [1, 2]. MAP3Ks, as serine/threonine kinases, are pivotal in this cascade, phosphorylating downstream MAP2Ks and MAPKs to engage in a wide variety of biological functions. These include critical roles in regulating cell apoptosis and autophagy, which are essential for basic bodily functions [3, 4]. Furthermore, MAP3Ks support embryonic development and contribute to sex determination [5, 6]. Additionally, the contribution of MAP3K4 to the development of other organs is highlighted, with its inactivation linked to a range of developmental anomalies, including skeletal disorders, neural tube closure defects and other malformation [7].

Similar to other components within the MAPK pathway, MAP3K4 functions as both a tumor promotor and suppressor, accelerating or decelerating tumor proliferation and metastasis [8]. This insight propels forward the search for targeted inhibitors that could be utilized in clinical trials aimed at treating cancers. However, the challenge of effectively targeting MAP3K4 to thwart tumor genetic activation and achieve therapeutic success remains a significant hurdle that demands more in-depth investigation.

In this review, we evaluated the physiological roles of MAP3K4 and the MAP3Ks family, with a particular emphasis on the complex roles of MAP3K4 and its involvement in the mechanisms of cancer cells.

## MAPK pathway and MAP3K4 gene

### MAPK transduction pathways

MAP kinase signaling pathways play an important role in regulating transcriptions, determining enzymatic efficiency, and phosphorylating components of the eukaryotic cytoskeleton [1]. The main MAPK modules consist of a series of kinases, including a MAPK that undergoes phosphorylation on threonine and tyrosine residues by an upstream MAPK kinase (MAP2K). Subsequently, the MAP2K is activated by an upstream MAP2K kinase (MAP3K) in a similar manner [2]. In another word, the entire MAPK signaling cascade operates through a three-tiered hierarchy of protein kinases: MAPKKKs (for instance, MAP3K4) at the apex, MAPKKs (such as MKK3/6 and MKK4/7) at the intermediary level, and MAPKs (like JNK and p38) at the foundation [9].

There are three distinct MAPK pathways: ERK [10], the p38 kinases [11], and JNK [12]. ERKs can be stimulated by various receptors, including cytokine receptors, heterotrimeric G-protein-coupled receptors, and growth factor receptor tyrosine kinases. The activation of p38 protein kinases occurs in response to cellular stressors such as lipopolysaccharide exposure, proinflammatory cytokines, and osmotic imbalance. JNKs are triggered via a variety of factors, such as cellular stressors like ultraviolet radiation, protein synthesis depressors and pro-inflammatory cytokines [1].

Mitogen-activated protein kinases (MAPKs) are a group of kinases significantly involved in many cancer processes. Mutations affecting essential cell signaling pathways are frequently linked with cancer [13].

### ***MAP3Ks family and MAP3K4 gene***

MAP3Ks, belonging to the serine/threonine kinase family, operate upstream of MAP2Ks and MAPKs, representing the most abundant type of MAPK signaling components in human cells. With approximately 24 identified members, ranging from MAP3K1 to MAP3K21, along with A-Raf, B-Raf, and C-Raf [14]. MAP3Ks are activated by upstream stimuli like inflammatory substances, antigens, toxins, and stressors, and connect the triggering of various MAPKs to the cellular response of each event [15]. Different MAP3Ks may activate various MAPK modules or activate the same unit but with varying timing or localization, producing alternative effects. Considering the broad spectrum of stimuli activating various MAPKs, the multitude of MAP3Ks serves as the primary mechanism for the attainment of diversity in MAPK activation [15, 16].

**Fig. 1:**
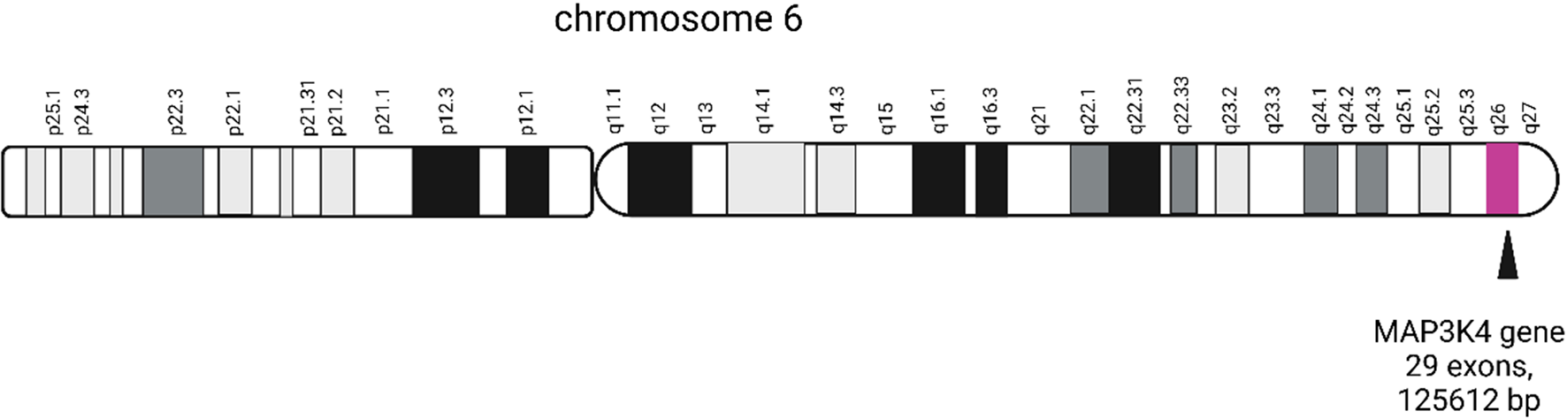
The detailed location of MAP3K4

The human MAP3K4 gene (GenBank: NM_005922.4), is located on chromosome 6q26, containing 29 exons, and 125,612 bp in length (Fig. 1. The detailed location of MAP3K4). The orthologs of human MAP3K4 gene are found in 534 organisms, such as zebrafish and the rhesus monkey. The human MAP3K4 protein (GenBank: NP_005913.3), co-localizing with Golgi-associated structures [17], is a 1608-amino acid (aa)-long protein with a molecular mass of 181,685 Da. It is also known as MEKK4 and MTK1.

### ***The downstream of MAP3K4***

**Fig. 2:**
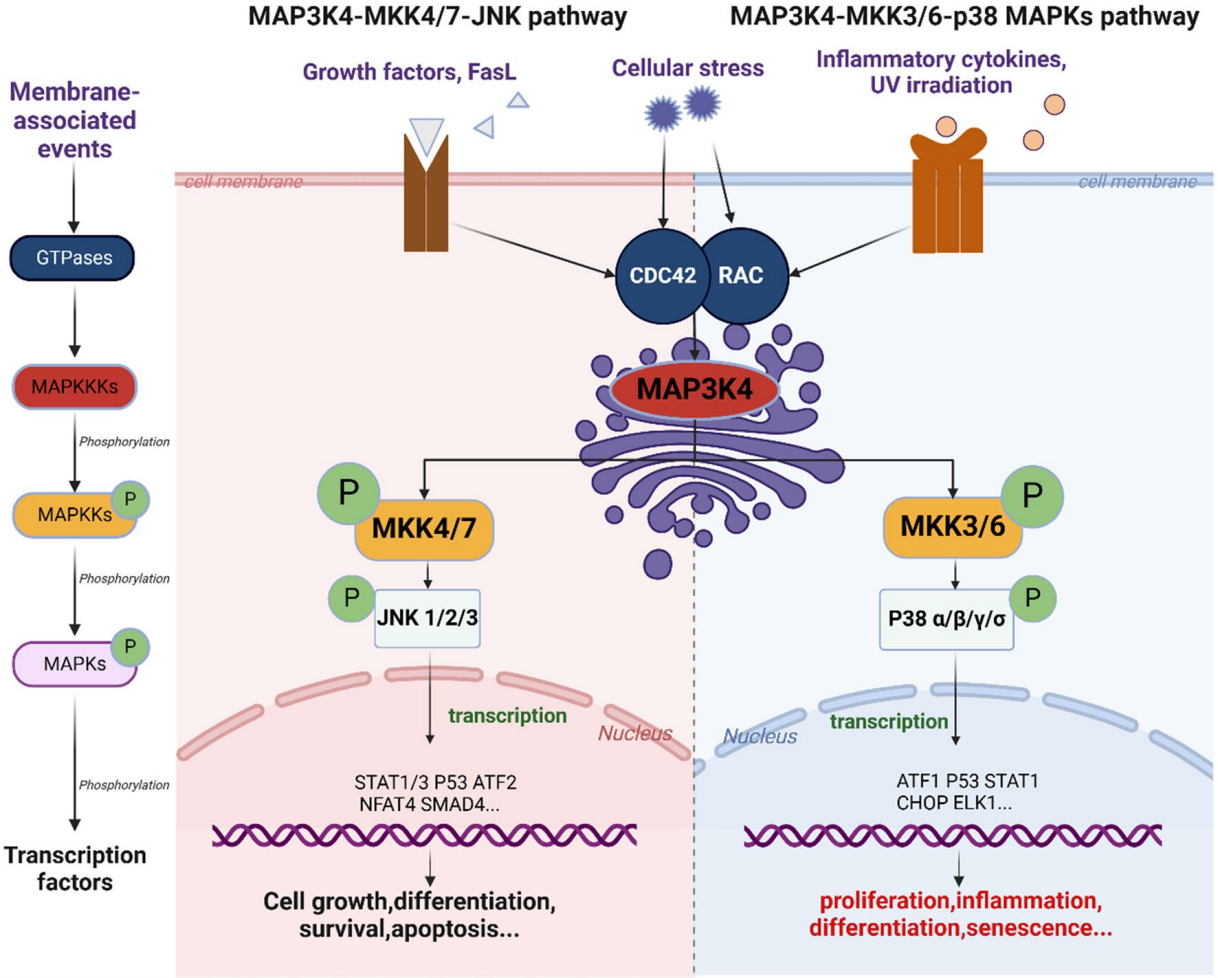
The MAP3K4 involved pathways. Extracellular stimuli (e.g., growth factors, UV) activate MAP3K4, co-localizing with Golgi-associated structures, through the mechanisms mediated by GTPases (RAC and CDC42). Once MAP3K4 is activated, it phosphorylates MAPKKs (MKK4/7, MKK3/6) and MAPKKs in turn phosphorylate MAPKs (JNK, p38). Lastly, activated MAPKs phosphorylate series of transcription factors like p53, thereby regulating gene transcription

As one of the MAP3Ks, MAP3K4 plays a crucial role in several MAPK pathways. Extracellular stimuli activate the MAPK pathways through mechanisms mediated by GTPases, RAC and CDC42 [17, 18]. It first phosphorylates two conserved serine (Ser) and threonine (Thr) regions on the enzyme activation loop of MKK3/6 and MKK4/7 [19]. Subsequently, MKK3/6 activates p38 through the activation loop by phosphorylating Thr and Tyr on the Thr-Gly-Tyr motif [20]. Regarding JNKs, Tyrosine 185 is preferentially phosphorylated by MKK4, while threonine 183 is preferred by MKK7, enabling the complete activation of JNKs [21] (Fig. 2. The MAP3K4 involved pathways).

Stimulated JNKs possess the capability to govern the transcription of the cMyc, p53, ELK1, ATF2, STAT1/3, and BCL2 family proteins. All of these proteins contribute to the management of numerous physiological events, which might include insulin signaling, neuronal activity, cell proliferation and apoptosis, immunological consequences [22, 23]. Similar to JNK activation, the p38 MAPKs govern cellular functions by promoting descending transcriptional targets such as PAX6, ETS1, AP-1, ATF1, and CHOP. The resulting proteins are responsible for transcription, chromatin remodeling, and gene regulation [14, 24].

Engagement of JNK and p38 MAPK pathways in normally functioning tissue is primarily caused by metabolic stress, DNA damage, cytokines, and growth factors. Both pathways exhibit altered protein expression in malignant cells, contributing either oncogenic or tumor-suppressive action [13, 25, 26].

## Cellular regulations and interactions of MAP3K4

### The regulation of cell apoptosis

MAP3Ks and the MAPK signaling pathway have been shown to be essential for bodily processes. MAP3Ks consist of an N-terminal domain, which lacks kinase function, and a C-terminal kinase domain. In contrast to full-length MAP3K1-3 (FL MAP3K1-3), the transmitting activation of FL MAP3K4 is hardly perceptible in most processes. MAP3K4 becomes an exceptionally powerful JNK activator when its N-terminal domain is removed, revealing that, unlike other MAP3Ks, MAP3K4's N-terminal domain hinders the enzyme's capacity to engage in MAPK signaling. Therefore, MAP3K4 initiation may occur when a molecule like DNA-damage-inducible 45 (GADD45) prevents the N-terminal domain from inhibiting the functional region anymore [3, 27]. The researchers came to the conclusion that MAP3K4's N-terminal domain incorporates an antiapoptotic promoting domain after noticing that MAP3K4 without N-terminal domain proved to be an extraordinarily effective apoptotic inducer, whereas FL- MAP3K4 failed to induce any apoptotic reactions and stimulated caspase-3-like proteases [3].

Stress can give rise to the formation of cytoplasmic stress granules (SGs), serving as a key adaptive defense mechanism by prohibiting the synthesis of misfolded proteins. RACK1, a structural transmitting protein, interacts with the MAP3K4, adapting to stress and facilitating type 2 stress to activate it; however, throughout type 1 stress, RACK1 is sequestered within SGs [28]. As a consequence, the first conditions prevent the second from triggering the MAP3K4-p38/JNK pathway and leading to apoptosis [29]. The phenomenon of chemotherapy-induced hypoxic resistance may be explored with the aforementioned results.

MAP3K4 can control apoptosis in addition to acting as a stimulating factor inducing apoptosis. For instance, experimental investigations have disclosed that a BRCA1-dependent, serum withdrawal-induced apoptotic pathway that incrementally encompasses H-Ras, MAP3K4, and JNK, subsequently followed by inducement of Fas ligand/Fas interactions and caspase-9-dependent signaling, resulting in the apoptosis of breast and ovarian malignancies [30].

### The regulation of cell autophagy

Autophagy is an evolutionarily stable metabolic mechanism, which entails the disintegration of organelles and cytosolic biomolecules. This mechanism is fundamental to cellular homeostasis, mammalian development, cancer, and immunology [31]. ATG5 and other autophagy-related proteins, conserved as significant regulators of autophagy, may be crucial in directing cell destiny [32]. AGT5 also takes involvement in the development of autophagosomes. It has been shown that the p38 MAPK is specifically concentrated on autophagosomes after being combined and activated by GADD45β. Active p38 phosphorylates ATG5 at threonine 75 at the autophagosomal membrane, which suppresses autophagy. In other words, this indicates that GADD45β-MAP3K4-mediated p38 activation selectively and spatially modulates ATG5 activity and adversely affects autophagosome maturation [33, 34].

The bacterial sensor NOD2 (nucleotide-binding, oligomerization domain 2) within the cells also initiates autophagy as a defense strategy against germs [35]. According to previous studies, NOD2 drives autophagy through a mechanism reliant on the activity of the receptor-interacting protein kinase 2 (RIP2). MAP3K4 and p38 serve as prerequisites for the induction of NOD2-dependent autophagy, which is hampered by collaboration with a PP2A phosphatase enzyme, alone with NOD2 and RIP2 [36]. Apart from bacteria and humans, autophagy can also interact with viruses. Viruses frequently take advantage of autophagy, a crucial phase for host immunity, for assistance in their survival. In DF-1 cells, GADD45β has been reported to form associations with MAP3K4 and its removal had an impact on the infection of the avian leukemia virus J (ALV-J). This indicates that ALV-J would potentially assist GADD45β in attaching to MAP3K4, enabling the p38 MAPK signaling pathway, which then inhibits autography [4].

### The interactions with binding partners

The Rho superfamily GTP-binding proteins CDC42 and RAC are widely known for their capacity to control cytoskeletal processes [37]. According to previous studies [1, 17], MAP3K4 combines with the two proteins, and a mutation of MAP3K4 without kinase activity precludes GTPase-deficient, activated CDC42 and RAC from triggering the JNK pathway. As a result, MAP3K4 is a leading contender to be a CDC42/RAC-regulated MAP3Ks. Given that GST-CDC42 binds to MAP3K4, generating this region in a GTP-dependent approach, and that MAP3K4 possesses a putative CRIB-like domain, it is likely that MAP3K4 belongs to the JNK pathway, which is governed by CDC42 and RAC [1].

Originally discovered as a part of the genes induced by inflammation and other biological stimulus, GADD45α is a tiny protein of 21 kDa that is regulated by p53. Since GADD45β and GADD45γ share a close relationship, they could possess similar but distinctive biological roles [27]. Through the utilization of yeast cells in an effective complementing examination procedure, growth arrest and GADD45 proteins have been demonstrated to be MAP3K4 activating agents. Studies came to the following conclusions: The GADD45 enzymes are MAP3K4 activating agents, adhering to its N-terminal part, and preventing the autoinhibitory region from blocking the kinase domain when they are attached to that area [38].

The multi-adaptor protein CIN85 is necessary for the continuous existence of neuronal cells as well as the reduction in the activity of tyrosine kinases in stimulated receptors [39]. Three SH3 domains in CIN85 distinctively attach the PxxxPR proline-arginine motif, which is present in a number of CIN85 effectors. The linkage of the SH3 domains of CIN85 to the special regions found within the MAP3K4 sequence causes MAP3K4 to become a new partner for CIN85. Researchers investigated the connection and discovered that this promotes the initiation of MKK6 and the cascade p38 MAP kinase in reaction to growth factor and oxidative stress stimulation. Furthermore, it has been demonstrated that MAP3K4's multi-ubiquitination and the activation of MAP3K4 by GADD45 proteins are controlled by CIN85 [40].

Axin is a multidomain protein that is fundamental and operates as a scaffold in Wnt signaling [41]. Axin binds to MAP3K1/4 before activating the p38/JNK MAPK downstream. MAP3K1 and MAP3K4 compete for Axin binding while adhering to separate locations, which implies that Axin could only bind to MAP3K1 or MAP3K4 based on particular signals or biological context [42]. Nevertheless, a number of Wnt pathway components, including CCD1, a known DIX domain protein crucial for Wnt, which control Axin's activation of the MAPK pathway [43]. When conjugated with CCD1, Axin is incapable of adhering to MAP3K1. Additionally, MAP3K4 and CCD1 physically cooperate in their physiological dimensions, blocking MAP3K4's capacity to bind to Axin [42].

TNF receptor-associated factor (TRAF) family proteins constitute a necessity for growth and immunity. TRAF proteins operate as scaffolding, which helps organize the signaling networks corresponding to particular binding sites in the plasma membrane. TRAF4 is an unique protein belonging to the TRAF family [44]. MAP3K4 binds the TRAF domain, and the activation of JNK by MAP3K4/ TRAF4 is prevented by the expression of the TRAF domain. Moreover, TRAF4 boosts MAP3K4 kinase activity by facilitating MAP3K4 oligomerization, and MAP3K4 dimerization may be chemically induced to enhance JNK activation. These outcomes demonstrate that TRAF4 mediates the JNK pathway through MAP3K4 [45].

A serine/threonine kinase termed GSK3 governs multiple signaling pathways. GSK3α and GSK3β are two isoforms that are very similar; the latter is a negative regulator of a variety of signaling networks in cells [46]. Given that it interacts with the kinase domain and disables dimerization and MAP3K4 activity, MAP3K4 is a MAP3K that GSK3β governs for its blockade of JNK and p38 signaling [47]. In contrast to TRAF4, Axin, and GADD45, GSK3 restricts MAP3K4 activity and prohibits it from activating JNK and p38.

Eukaryotic cells are capable of rapidly identifying and responding to changing environmental conditions to grow and thrive. This is normally accomplished through the activity of plasma membrane-based sensor proteins that are connected to signaling pathways for mitogen-activated protein (MAP) kinase. Osmotic stress leads to the breakdown of the actin cytoskeleton, a cessation of the cell cycle, and a boost in the excessive osmolarity growth mitogen-activated protein kinase pathway. According to studies, the MAP3K4 homolog Ssk2p manages the actin cytoskeleton's recovery from osmotic stress.

The examination demonstrates that the mechanisms are conserved, thus MAP3K4 can also express itself at polarized growth locations, establish an intricate network with actin, and correct the actin recovery defects in osmotically challenged yeast cells. In addition, by functioning upstream of tropomyosin and downstream of Bud6p and Pea2p, Ssk2p facilitates actin recovery, possibly through enhancing the formins' actin nucleation activity [48].

**Fig. 3:**
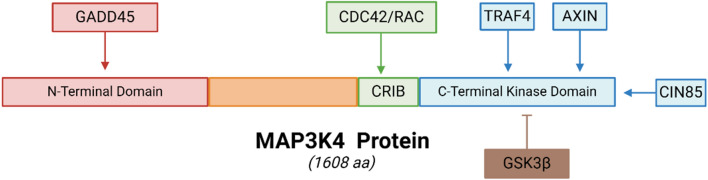
The functional domain of MAP3K4 protein and the interactions with binding partners. A schematic diagram for human MAP3K4 protein structure is shown. It is a 1608-amino acid (aa)-long protein with two significant domains and interactions. The N-terminal domain without kinase function hinders the enzyme's capacity, unless a molecule like GADD45 attaches to this domain could initiate MAP3K4 function. The C-terminal kinase domain is the region that interacts with other important molecules, like CIN85 and AXIN, and performs functions. CDC42/RAC interactive bingding (CRIB) domain is encoded just upstream of the catalytic domain to combine with the two proteins

Together, the detailed interactions among MAP3K4 and other molecules can be seen in Fig. 3 (The functional domain of MAP3K4 protein and the interactions with binding partners).

## The role of MAP3K4 in governing growth and development of body

### ***The linkage between MAP3K4 and embryo development***

To accurately work toward pre-implantation advancement, researchers chose P19 embryonal carcinoma cells, a viable test tube for mouse pre-implantation gestation [49]. Through the application of optimum cultivation conditions and a stimulation agent, P19 cells can generate the three germ layers: endoderm, mesoderm, and ectoderm [50].

Retinoic acid can induce the expression of Ga13 and the activation of JNK in P19 cells, leading to the development of primitive endoderm [51]. In the process controlling the differentiation of P19 stem cells into primitive endoderm, subsequent investigations have identified MAP3K4 as upstream of JNK [52].

An essential phase in the embryo implantation process is embryo adhesion. For embryo adhesion, homeobox A10 (HOXA10), a genetic mediator of endometrial receptivity, is indispensable. MAP3K4 phosphorylates HOXA10 at threonine 362, boosting the bonding between the embryo and endometrial epithelium, alone with the HOXA10-regulated transcriptional recreations. In endometrial epithelial cells, specific MAP3K4 deletion or kinase inactivation reduces embryo-epithelial adhesion. Thus, the recently identified function of MAP3K4 as an entirely novel physiologically supportive regulator of HOXA10 initiation provides valuable information to maximize the success of embryo implantation [53].

After adhesion, the embryo begins to develop. During the formation of the embryo, the epithelial-mesenchymal transition (EMT) developmental scheme provides the basis for the proper construction of the body plan and the differentiation of many tissues and organs [54]. When trophoblasts differentiate during placentation, EMT occurs with a degradation of E-cadherin and the establishment of trophoblast invasiveness [55]. Researchers uncovered that MAP3K4 expression significantly decreases during differentiation yet prominently expressed in the developing embryo, trophoblast stem (TS) cells, and cells derived from TS cells. They noted that MAP3K4 modulates differentiation, holds back devotion to an invasive phenotype, and preserves TS cells in addition to fibroblast growth factor 4 (FGF4). Firstly, reduced MAP3K4 kinase activity in TS cells precludes FGF4 from initiating JNK and p38, contributing to a drop in E-cadherin expression and a spike in invasiveness, and the synthesis of chemicals that promote TS cell progression toward the spongiotrophoblast and syncytiotrophoblast lineages [56]. Secondly, to facilitate the acetylation of histones H2A and H2B (H2A/H2BAc) on the promoter sequences of genes essential for preserving the epithelial phenotype, MAP3K4 activates CBP, a histone acetyltransferase (HAT). As a result, the combined loss of MAP3K4/ CBP activity represses expression of epithelial genes and causes TS cells to undergo EMT while maintaining their self-renewal and multipotency properties [6]. Thirdly, MAP3K4 expression hampers HDAC6 transcription and activity, which serves as a prerequisite for deacetylating cytoplasmic and nuclear proteins vital to EMT. HDAC6 deacetylates the promoter regions of the genes that code for proteins that make up tight junctions, which reduces cell–cell adhesion and is a hallmark of the mesenchymal phenotype. HDAC6 expression and activity are both elevated in TS cells with the loss of MAP3K4 operation, and HDAC6 is silenced to restore epithelial characteristics [57]. MAP3K4 collectively oversees the fundamental selection of TS cells to either engage in renewal for stem cell maintenance or initiate a program of increased motility, invasiveness, and differentiation for placental development.

Fetal development instability exerts profound adverse effects on human wellness, worsening fetal and infant morbidity and mortality while raising the likelihood of lifelong health issues. Fetal growth restriction (FGR) or intrauterine growth limitation, for short, is the inability of a human being to reach their genetically assigned size as a consequence of placental insufficiency [58]. MAP3K4 stimulates CBP and suppresses HDAC6 to directly enhance insulin-like growth factor 1 receptor (IGF1R) transcript levels. IGF/insulin signaling plays an essential role in fetal growth, and mice with altered IGF/insulin signaling have been used in thoroughly researched models of FGR [59]. In TS cells and the placenta, MAP3K4 is essential for the IGF1R/IR and Akt signaling pathways, and MAP3K4 kinase inactivation causes FGR driven by placental insufficiency [60].

### ***The decisive role of MAP3K4 in sex determination***

The Y-linked gene SRY's existence or absence defines the sex of mammals. Sex-determining region of the Y (SRY) protein is responsible for boosting the expression of a related gene, SOX9, acting as a mediator of transcription, in the developing male (XY) gonad. This procedure leads to the initiation of the maturation of the testis while simultaneously repressing the growth of the ovary. In the human population, sex-related disorders can range in severity from minor genital abnormalities to gonadal sex reversal [5]. The recessive boygirl (byg) mutation was discovered in some individuals in question. The MAP3K4 gene has undergone through an A to T transversion mutation, resulting in an early stop codon. The detection of both active MKK4 and p38 in the coelomic region of the XY gonad at 11.5 days post also confirmed the connection between MAPK signaling in developing gonadal somatic cells and the regulation of SRY expression [61]. In the meantime, GADD45γ genetically interacts with MAP3K4 during sex determination, participating in promoting MAP3K4-mediated activation of p38 MAPK signaling in embryonic gonadal somatic cells [62].

T-associated sex reversal is a unique occurrence on proximal mouse chromosome 17. In B6-YAKR embryos with the Thp deletion, experiments proved that SRY expression is disrupted, which causes XY gonadal malformations. These abnormalities are corrected by the properly functioning MAP3K4 bacterial artificial chromosome via restoring the normal SRY sequence. These findings indicated that MAP3K4 level is a key driver of SRY and that MAP3K4 haploinsufficiency is a contributory factor of T-associated sex reversal [5]. All of these data strengthen the fact that MAP3K4-dependent signaling events are absolutely essential for allowing SRY to express appropriately as the testis matures.

### ***The auxiliary function of MAP3K4 in the growth of other organs***

Clinically vital human abnormalities, such as skeletal disorders and neural tube closure issues, are currently under investigation by researchers in order to understand the signaling pathways involved. MAP3K4-regulated p38 activity is critical for neurulation. Targeted mutation of the active site lysine of MAP3K4 results in a kinase-inactive MAP3K4 protein (MAP3K4K1361R). Heat shock-induced actin cytoskeleton instability and a significant reduction in the phosphorylation of p38 and HSP27 have been observed in MAP3K4K1361R fibroblasts. Homozygous for this genetic variation leads to embryos perish before birth due to skeletal abnormalities and neural tube issues [63]. Neural tube abnormalities (NTDs) in MAP3K4-MKK4-p38/JNK-deficient mice are strongly penetrant and cannot be reversed by folic acid or inositol supplementation. Furthermore, during neural tube growth, MAP3K4 is vital in controlling MKK4 effectiveness and apoptosis, with the emerging neuroepithelium expressing high levels MAP3K4. Neural tube abnormalities (NTDs) that are strongly penetrant in MAP3K4-MKK4-p38/JNK-deficient mice cannot be reversed by folic acid or inositol supplementation [64]. Periventricular heterotopia (PH), a brain abnormality, can be recognized by neuronal nodules that are irregularly positioned along the cerebral cortex's lateral ventricles. Human mutations in the Filamin A (FLNA) or ADP ribosylation factor guanine exchange factor 2 (ARFGEF2) genes can lead to PH. Researchers established a causal link between MAP3K4 and FLNA that affects the initiation of neuronal migration and provides insight into the pathogenesis of human PH [65].

As a molecular entity that is intimately linked to MAP3K4, GADD45 participates in signaling pathways that control organs. Valproic acid (VPA), a mood stabilizer and anticonvulsant, can upregulate GADD45 by activating MAP3K4, and subsequently drive the downstream JNK cascade, regulating neurite outgrowth [66]. GADD45α is also expressed in the developing cortices of mice and humans. Its overexpression alters the morphology of neurons, decreases neurite complexity, induces soma hypertrophy, and accelerates cell death, while the reduction of both GADD45α and MAP3K4 hinders neurite outgrowth. Therefore, the expression of GADD45α and MAP3K4 in the normal, developing brains are tightly regulated, and factors altering their expression may have a significant impact on how neural circuitry develops [67]. Moreover, glucocorticoid and JNK signaling are essential for the arrangement of mammary epithelial cells into 3D acinar structures, and the latter is triggered by BRCA1-GADD45β- MAP3K4 [68]. Ultimately, researchers found that, under the guidance of differentiation-promoting cytokine receptor signaling, GADD45γ is a critical educator of long-term repopulating hematopoietic stem cells (LT-HSC) differentiation. By predominantly activating MAP3K4-mediated MAPK p38, GADD45γ promotes and expedites LT-HSC development, bypassing the auto-renewal program [69]. Except for these detailed investigations, MAP3K4 has been demonstrated to be linked with intellectual development [70], innate immune response [71], inner ear morphogenesis [72], production of the cushion mesenchyme for heart development [73] and craniofacial development [74], all of them are of critical significance in the course of typical growth and development.

## The complex function of MAP3K4 concerning tumor

### Working as a tumor-promotor

An analysis of whole genome sequencing (WGS) data and whole exome sequencing (WES) data reported a finding: Non-coding mutations have the capacity to generate splice sites, and certain mutation-induced splicing modifications occur in tumor-related genes like MAP3K4 and TP53, frequently resulting in truncated proteins and influencing gene expression. To avoid overlooking these mutations based on conventional annotations, our researchers should concentrate more on the potential for splicing modifications in non-coding mutations in cancer studies and the oncogenic impacts of splice creating non-coding mutations [75].

In response to HER2/HER3 signaling, MAP3K4 is essential for breast cancer cells to migrate and generate extracellular acidification [76]. Actin polymerization and intracellular actin-binding protein coordination are necessary for cell migration, which is a crucial component of cancer cell invasion and metastasis [77]. MAP3K4 interacts with actin through a 40 amino acid region known as the actin interacting region (AIR). The constitutive association between MAP3K4 and actin is disrupted by the deletion of the AIR in MAP3K4. These exhibit the close proximity of MAP3K4 to the actin cytoskeleton. What’s more, MAP3K4 is paired with G-protein-coupled receptor 2 interacting protein 1 (GIT1) and constitutes vital components for acidifying the extracellular space [76]. Matrix metalloproteinases and other enzymes required for the migratory response apparently benefit from the extracellular environment’s acidity, which maximizes their enzymatic activity [78].

Small non-coding RNAs, called microRNAs (miRNAs), silence genes by post-transcriptionally attaching to the 30-UTR of their intended target mRNAs [79]. One of the primary mechanisms for regulating gene expression has been considered to be co-expression network analysis of miRNA and mRNA. Since miR-24-1 targets genes on critical signaling pathways that are closely related to cell proliferation and migration, it is conceivable that miR-24-1 promoted tumor progression by regulating the expression of the MAPK pathway in ependymal tumor cells [80].

As a member of the galectin family, human galectin-1 exhibits significant expression various malignancies and is involved in numerous oncogenic processes. Galectin-1 contributes to the MAP3K4-JNK-AP1 transduction mechanism, emphasizing the crucial role of MAP3K4 in this process [8].

### Working as a tumor-suppressor

Larger tumor size, vascular invasion, intrahepatic spreading, and lymph node metastasis, all indicators of a poor prognosis and recurrence of tumor were substantially associated with low expression of MAP3K4 [81]. For instance, the putative tumor suppressor MAP3K4 potently controls the EMT process and invasive growth in intrahepatic cholangiocarcinoma (iCC), despite being highly down-regulated and frequently mutated [82]. One prospective strategy that offers an unconventional approach to mitigate malignant tumor is to focus on the altered metabolic state reconfigured by oncogenic transformation. For example, the TNF and MAP3K4-p38Noxa pathways are substantially activated in cystine-dependent breast cancer cells and tumors, rendering them vulnerable to the necrosis caused by cystine deprivation [83].

It has been established that lncRNAs play an enormous part in the regulation of MAPKs in the growth of cancer [84]. A recently discovered lncRNA, GATA binding protein 6 antisense (GATA6-AS), is downregulated in the tumor tissues of cervical squamous cell carcinoma patients. According to this study, MAP3K4 is downregulated when GATA6-AS is expressed, hindering cancer cells from migrating and invading [85]. With a similar mechanism, the long non-coding RNA-OIS1 inhibits HPV-positive, but not HPV-negative cervical squamous cell carcinoma by upregulating MAP3K4 [86]. Downregulation of ERBB3 diminishes cervical cancer cell motility, proliferation, and invasion via its interaction with MAP3K4, which still controls the occurrence and development of the disease [87]. Similarly, the overexpression of miR-148a significantly inhibits cutaneous squamous cell carcinoma (CSCC) cell proliferation and metastasis by down-regulation of MAP3K9 and MAP3K4 expression [88].

CircRNAs also play an indispensable supervisory role in tumor formation and carcinogenesis of tumors, as evidenced by mounting research. Researchers revealed that the phosphorylation of eukaryotic translation initiation factor 2 (eIF2) and the formation of SGs are suppressed due to circRNA-CREIT's promotion of the degradation of PKR through the HACE1-mediated ubiquitin–proteasome pathway. CircRNA-CREIT's suppression of SGs formation allows more RACK1 protein to engage with MAP3K4 and activate the apoptotic pathway [89]. Furthermore, it appears that apoptosis pathway plays a significant role in suppressing tumor development [30].

**Fig. 4:**
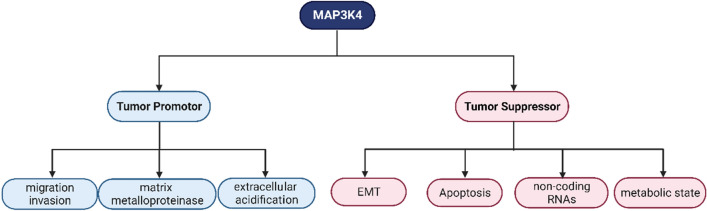
The dual role of MAP3K4 in tumorigenesis. MAP3K4-dependent cellular processes can be classified on the basis of the role they play during tumorigenesis

Together, the dual role of MAP3K4 and the detailed mechanisms regulating tumors can be seen in Fig. 4 (The dual role of MAP3K4 in tumorigenesis) and Table 1.

**Table 1:** The detailed mechanisms of MAP3K4 regulating malignant cancers

Cancer type	Outcomes	Mechanisms
Breast cancer	Promoting cancer cells invasion and metastasis	Regulating actin cytoskeleton and extracellular acidification
	Rendering cancer cells vulnerable to the necrosis	Targeting the modified metabolic state through the TNF and MAP3K4-p38Noxa pathways
	Suppressing cancer drug-resistance	CircRNA-CREIT and MAP3K4 opening the apoptotic pathway
Bladder cancer	Promoting cancer cells invasion	Galectin-1 regulating MMP9 via Ras-Rac1- MAP3K4-JNK-AP1 signaling pathway
Intrahepatic cholangiocarcinoma	Suppressing cancer invasive growth	Controlling the EMT process
Cervical squamous cell carcinoma	Hindering cancer cells from migrating and invading	GATA6-AS inhibiting the expression of MAP3K4
	Inhibiting HPV-positive, cancer cells	Long non-coding RNA-OIS1 upregulating the expression of MAP3K4
	Diminishing cancer cells motility, proliferation, and invasion	Downregulating the interaction between ERBB3 and MAP3K4
Ependymal tumor	Promoting tumor progression	MiR-24–1 regulating the expression of the MAPK pathway
Cutaneous squamous cell carcinoma	Inhibiting cell proliferation and metastasis	MiR-148a down-regulating MAP3K9 and MAP3K4 expression

### Working as a biomarker

The identification and characterization of biomarkers in tumor biopsies from patients with known clinical responses, both before and after therapy, could assist in identifying the patients who respond poorly. This allows for the selection of more efficient treatment, refraining from overtreatment, and subsequently lowering associated medical expenses [90].

A tumor is considered clinically radio-resistant when radiation fails to shrink its size or when a recurrence happens following a potential regression [91]. In patients receiving radiation, the MAP3K4 gene is discovered to accurately and independently predict distant metastasis free survival (DMFS). Due to a negative interaction between high MAP3K4 expression and radiotherapy, patients with excessive expression of MAP3K4 receiving radiotherapy (RT) exhibit shorter DMFS than patients with low expression of MAP3K4. Identifying biomarkers indicative of the initial response to preoperative radiotherapy (pRT) would be advantageous for forecast the clinical outcome in radiotherapy treated patients [92].

MAP3K4 is a specific gene related to tumorigenesis. Its mutation, regulating filamin expression and neuronal migration [7], was detected in breast neuroendocrine neoplasms (NENs), a peculiar histologic subtype [93]. Genome-wide sequencing shows that MAP3K4 and its mutations also frequently appear in several kinds of tumor tissues, such as serous endometrial tumor [94], wilms tumor [95], EBV-positive gastric tumor [96], pancreatic cancer [97], and family lung cancer [98].

### ***Targeting MAP3K4 as cancer therapy***

ERKs, JNKs, and p38 MAPKs proteins are part of the MAPK family, each protein plays distinct roles in either fostering or restraining cancer. As these three MAPK families are tightly regulated by the same upstream substances, which are made up of MAPKK and MAPKKK, selectively activating tumor-suppressor without simultaneously stimulating the promotor has become a challenging issue. This presents an innovative opportunity for the research and development of novel anti-cancer drugs.

Triptonide, a component isolated from the traditional Chinese herbal, exhibits an extensive spectrum of biological characteristics [99]. Triptonide potently suppresses expression of the cell cycle critical gene CDK3 while selectively activates the MAP3K4-MKK4-p38 signaling pathway and promotes expression of p38 downstream tumor suppressor p21 [100]. This leads to cancer cell cycle arrest at the G2/M phase, causing a potent anti-cancer impact, especially in the treatment of pancreatic cancer and contributing to the development of new cancer therapies [101].

Similarly, dehydroeffusol, an active component isolated from a medicinal herb named Juncus effuses, is explored as a novel anti-gastric cancer agent. It activates the intracellular tumor-suppressive stress response via upregulating the critical ER stress marker DNA damage-inducible transcript 3 (DDIT3) through the regulation of activating transcription factor 4 (ATF4). This component significantly triggers the MAP3K4-MKK3/6-p38-DDIT3 stress response transmission cascade while mildly dampening ERK signaling. Dehydroeffusol, with the goal of mitigating gastric cancer cell proliferation and tumorigenicity, selectively exhibits an overwhelming tumor-suppressive endoplasmic reticulum (ER) response to stressful conditions and a moderate apoptotic reaction [102].

In addition to the traditional tumor-targeting medications, researchers priortize on investigating brand-new medications with remarkable preventive efficacy. For instance, the Chinese herbal combination Antitumor B (ATB), which contains six distinct species of plants, regulates genes involved across multiple cellular signaling networks, including MAP3K4. ATB treatment drastically reduces lung tumor multiplicity and tumor load in mice of different genotypes, indicating that it is an efficient chemopreventive against mouse lung carcinogenesis [103].

MAP3K4 plays significant roles in cancers, but it doesn’t only depend on itself rather relies heavily on the entire pathways. Therefore, the listed Chinese herbal components selectively activate the MAP3Ks–MAP2Ks-MAPKs signaling pathway rather than just MAP3K4 solely. What’s more, we need to pay much attention to target other molecules downstream the p38/JNK pathways, thus limiting the response to only a subset of p38/JNK-mediated responses, thereby ensuring that only certain functions are eliminated or initiated [19, 104].

## Discussion

Since its initial discovery in the 1990s, much progress has been made in understanding of the regulation and functions of MAP3K4. Researchers have comprehended its connection with the classical MAPK pathways, mastered its functions in regulating normal growth and development of the human body, investigated its interaction with other key molecules inside cells, and explored its complex role in governing malignant carcinomas. Nevertheless, comprehension of several fundamental features of MAP3K4's functionality is currently limited. For instance, the search for novel medications that selectively target MAP3K4 persists to be challenging.

A deeper understanding of the MAP3K4 mechanism will give rise to better therapeutic strategies for cancer therapy with higher favorable outcome rates. It’s long been known that MAPK pathways play dual roles in regulating tumor progression. While medicines based on MAP3K4 inhibition- effectively halt tumor growth, it should be noted that they may also make it easier for malignancies to occur in other tissues exposed to oncogenic triggers.

MAP3K4 is a part of the MAPK pathways, which are crucial for transmitting signals from the outside of cells to the inside, affecting various cellular processes like growth, differentiation, and apoptosis [1]. This process consists of two steps. First, how the functions of MAP3K4 initiate based on the balance of extracellular signals. Second, how the roles of MAP3K4 change in cellular signaling pathways. So far, research has only reported that MAP3K4 activity is stimulated or suppressed by extracellular signals like growth factors, inflammatory cytokines, and environmental stress [47]. But almost no researchers have explored the specific molecular mechanisms through which these factors affect MAP3K4, thereby activating or inhibiting the entire MAPK pathways. When it comes to the “roles change” inside cells, it may imply that the roles of MAP3K4 in the cell can shift from being beneficial to harmful. In most cases, it promotes growth and survival, while its same involving signaling pathways can also lead to uncontrolled cell growth even malignant diseases under certain conditions. This concept highlights the complexity of signaling pathways and the importance of maintaining a proper balance of signals for cellular health.

The connection between MAPK signaling pathways and other cellular pathways or components shouldn’t be ignored. MAP3K4 and the entire MAPK signaling pathways are truly responsible for delivering extracellular stimuli, but they are not isolated in vivo. Indeed, they are capable of activating or inhibiting other enzymes and consequently regulating a broad range of physiological responses. Besides being able to act as promotors or suppressors in cellular functions, they can also serve as signal transduction mediators, responsible for signal transmit among complex pathway networks [105]. Downstream signaling pathways are quite divergent and different pathways may interact with one another to coordinate cellular processes. This concept guides us that future investigations would benefit from attention to the interaction between different pathways, and the coordination among signaling events.

The MKKKs can serve as candidates for the creation of inhibitors that prevent MAPK from being inhibited universally by preventing stimulus-specific MAPK-dependent cell activity. Human conditions are crucial and necessitate comprehensive investigation into the inducible gene transcription regulated by s MKKKs before having the capacity to focus on MKKKs to precisely inhibit the intended genes and behaviors.

## Data Availability

Not applicable.

## Abbreviations

MAP3K4: Mitogen-activated protein kinase kinase kinase-4
MAP: Mitogen-activated protein
GADD45: Growth arrest and DNA-damage-inducible 45
TRAF: TNF receptor-associated factor
SGs: Stress granules
RIP2: Receptor-interacting protein kinase 2
ALV-J: Avian leukemia virus J
HOXA10: Homeobox A10
EMT: Epithelial-mesenchymal transition
FGF4: Fibroblast growth factor 4
TS: Trophoblast stem
FGR: Fetal growth restriction
IGF1R: Insulin-like growth factor 1 receptor
SRY: Sex-determining region of the Y
byg: Boygirl
HSP27: Heat shock protein 27
NTDs: Neural tube abnormalities
PH: Periventricular heterotopia
FLNA: Filamin A
ARFGEF2: ADP ribosylation factor guanine exchange factor 2
VPA: Valproic acid
LT-HSC: Long-term repopulating hematopoietic stem cells
DMFS: Distant metastasis free survival
pRT: Preoperative radiotherapy
NENs: Neuroendocrine neoplasms
AIR: Actin interacting region
GIT1: G-protein-coupled receptor2 interacting protein 1
MMP9: Modifying matrix metalloproteinase-9
iCC: Intrahepatic cholangiocarcinoma
GATA6-AS: GATA binding protein 6 antisense
CSCC: Cutaneous squamous cell carcinoma
ER: Endoplasmic reticulum
WGS: Whole genome sequencing
WES: Whole exome sequencing
eIF2: Eukaryotic translation initiation factor 2
DDIT3: DNA damage-inducible transcript 3
ATB: Antitumor B
